# Will initial consultation patterns among undiagnosed cancer patients be the same after this COVID-19 pandemic? Experiences from the 2011 triple disaster in Fukushima, Japan

**DOI:** 10.7189/jogh.10.020343

**Published:** 2020-12

**Authors:** Akihiko Ozaki, Toyoaki Sawano, Hiroaki Saito, Tetsuya Tanimoto, Masaharu Tsubokura

**Affiliations:** 1 Department of Breast Surgery, Jyoban Hospital of Tokiwa Foundation, Iwaki, Fukushima, Japan; 2 Department of Surgery, Sendai City Medical Center, Sendai Open Hospital, Sendai, Miyagi, Japan; 3 Department of Gastroenterology, Sendai Kousei Hospital, Sendai, Miyagi, Japan; 4 Medical Governance Research Institute, Minato-ku, Tokyo; 5 Department of Radiation Health Management, Fukushima Medical University, Fukushima, Japan

The novel severe acute respiratory syndrome coronavirus 2 (SARS-CoV-2), which causes the coronavirus disease 2019 (COVID-19), first broke out in Wuhan, China in December 2019, and has spread out worldwide causing a global pandemic. As cancer patients are deemed to be a high-risk population of COVID-19, the effect of COVID-19 on the cancer population and their treatment has already been widely discussed across various media and academic journals [1-3]. Briefly, it is important not only to protect cancer patients from SARS-CoV-2 infection, but also to continuously provide appropriate and timely care depending on the urgency of their conditions. However, such discussions have primarily focused on patients already diagnosed with cancer – patients waiting for treatment inception, patients already receiving treatment, and survivors in surveillance after treatment, and the short-term health effects among these populations. In contrast, little has been said regarding undiagnosed cancer patients (ie, before cancer diagnosis) and/or their long-term health consequences.

When considering undiagnosed cancer patients during the COVID-19 pandemic, we believe that our experiences in Fukushima would help further develop our discussions. We have been involved in care and research on cancer patients in Fukushima following the 2011 triple disaster (earthquake, tsunami, and nuclear accident) of the Great East Japan Earthquake, witnessing how long-term health effects have developed since the onset of this complex disaster. Arguably, the radiation disaster and the pandemic would indeed be seemingly different; however, COVID-19 is caused by an intangible pathogen with relatively unknown effects, which has some similarities with radioactive substances in their influence on human behavior as both would make people isolate themselves from others. For example, in the coastal area of Fukushima, the fear of radiation exposure has persistently prevented local residents from consuming locally grown food for more than five years after the disaster, although the air radiation dose rate was demonstrated to be at an acceptable level just a few years following the disaster [4]. This means that people may change their behavioral patterns for much longer than reasonably anticipated.

In the wake of the triple disaster, the most striking observation regarding cancer was an increase in the proportion of undiagnosed symptomatic breast cancer patients in the affected areas who delayed seeking initial medical consultation, and this situation has persisted for over five years after the disaster [5]. More concretely, 18.0% of the post-disaster patients delayed their initial medical consultation for 12 months or longer, while such a delay had occurred only 4.1% of the pre-disaster population (age adjusted risk ratio post- vs pre-disaster: 4.49, 95% confidence interval 1.73-11.65) [5]. Further, there is anecdotal evidence that the similar delay occurred among undiagnosed symptomatic colorectal cancer patients in the post-disaster period [6]. The most likely explanation for this is a decreased interest in seeking medical consultation for apparently non-urgent symptoms, as suggested by our in-depth interviews with some of the patients [6,7]. What should be noted is that these post-disaster changes in behavioral patterns among undiagnosed symptomatic cancer patients occurred although the local cancer care had been restored to enable the post-disaster cancer patients to receive timely initial treatment, at least six months after the disaster [8].

Similarly, when the “New Normal” is established during the COVID-19 pandemic, we are concerned that initial medical consultation among undiagnosed symptomatic cancer patients could be delayed because of the hesitance to request medical consultation promptly for fear of contracting COVID-19. Given that the current pandemic is projected to last until 2022 [9], and that this projection has been repeatedly disseminated via various media outlets, baseline motivation for medical consultation for non-urgent symptoms may decline for the next several years because of fear of infection. The same may be true with cancer screening for asymptomatic populations, given that the priority of such preventive measures may further decline in the same context.

**Figure:**
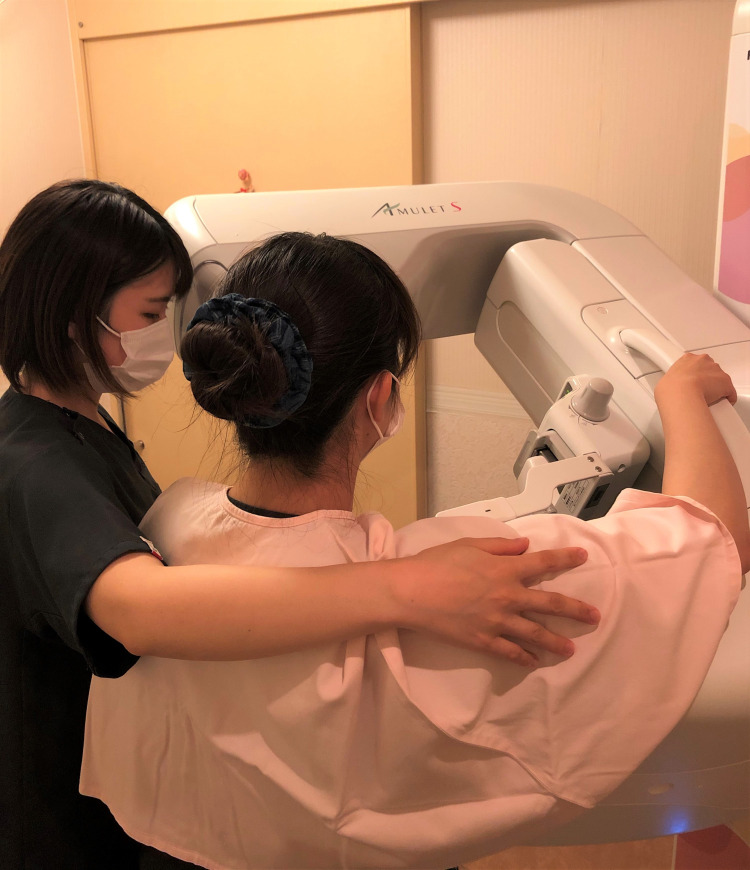
Photo: from the Jyoban Hospital of Tokiwa Founation, used with permission.

Naturally since its outbreak, COVID-19 has drawn huge attention to its direct health effects and interrupted care for other conditions including cancer, as originally seen in the 2011 triple disaster in Fukushima. However, once its death toll declines and people become accustomed to the New Normal soon, an interest in the COVID-19 and its related health problems would potentially diminish all in academic, mass media and general communities, as seen following the triple disaster. In this regard, health care professionals, public health practitioners, and policy makers should have a long-term perspective to reveal and mitigate potentially diverse and long-term health effects on cancer caused by the COVID-19 pandemic. To this end, appropriate designing of researches to clarify these health issues and relevant logistics enabling such researches are both essential. In the meantime, it is important to enhance awareness of signs and symptoms suggestive of cancer among the general public and to motivate them to regularly participate in cancer screening programs and/or do appropriate self-examination and self-care. Both in research and clinical practice, cross-sectional and longitudinal collaborations involving multiple stakeholders are critical to protect health and well-beings of cancer patients in the long term. Specifically, a close attention should be paid to whether a proportion of undiagnosed symptomatic cancer patients that delay seeking health care would be increased for fear of contracting COVID-19, a lesson learned from the 2011 triple disaster that should be shared globally.
